# A Multi-center Study on the Reproducibility of Drug-Response Assays in Mammalian Cell Lines

**DOI:** 10.1016/j.cels.2019.06.005

**Published:** 2019-07-10

**Authors:** Mario Niepel, Marc Hafner, Caitlin E. Mills, Kartik Subramanian, Elizabeth H. Williams, Mirra Chung, Benjamin Gaudio, Anne Marie Barrette, Alan D. Stern, Bin Hu, James E. Korkola, LINCS Consortium, Joe W. Gray, Marc R. Birtwistle, Laura M. Heiser, Peter K. Sorger

**Affiliations:** 1 Laboratory of Systems Pharmacology, HMS LINCS Center, Harvard Medical School, Boston, MA 02115, USA; 2 Department of Pharmacological Sciences, Drug Toxicity Signature Generation (DToxS) LINCS Center, Icahn School of Medicine at Mount Sinai, One Gustave L. Levy Place, Box 1603, New York, NY 10029, USA; 3 Microenvironment Perturbagen (MEP) LINCS Center, OHSU Center for Spatial Systems Biomedicine, Oregon Health & Sciences University, Portland, OR 97201, USA; 4 Present address: Ribon Therapeutics, Inc, 99 Hayden Avenue, Lexington, MA 02421, USA; 5 Present address: Department of Bioinformatics & Computational Biology, Genentech, Inc, South San Francisco, CA 94080, USA; 6 Present address: Department of Chemical and Biomolecular Engineering, Clemson University, 206 S. Palmetto Blvd., Clemson, SC 29634, USA; 7 Present address: Department of Data Sciences, Dana-Farber Cancer Institute, Boston, MA 02115, USA; 9 Lead Contact

## Abstract

Evidence that some high-impact biomedical results cannot be repeated has stimulated interest in practices that generate findable, accessible, interoperable, and reusable (FAIR) data. Multiple papers have identified specific examples of irreproducibility, but practical ways to make data more reproducible have not been widely studied. Here, five research centers in the NIH LINCS Program Consortium investigate the reproducibility of a prototypical perturbational assay: quantifying the responsiveness of cultured cells to anti-cancer drugs. Such assays are important for drug development, studying cellular networks, and patient stratification. While many experimental and computational factors impact intra- and inter-center reproducibility, the factors most difficult to identify and control are those with a strong dependency on biological context. These factors often vary in magnitude with the drug being analyzed and with growth conditions. We provide ways to identify such context-sensitive factors, thereby improving both the theory and practice of reproducible cell-based assays.

## In Brief

Factors that impact the reproducibility of experimental data are poorly understood. Five NIH-LINCS centers performed the same set of drug-response measurements and compared results. Technical and biological variables that impact precision and reproducibility and are also sensitive to biological context were the most problematic.

## Graphical Abstract

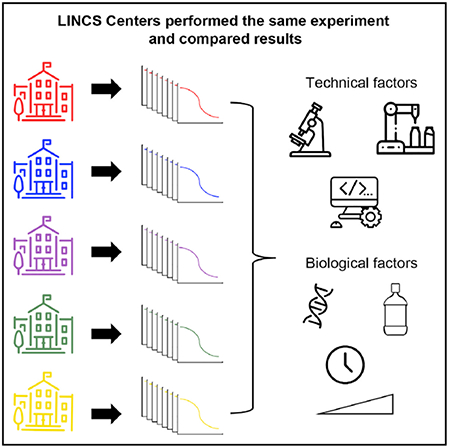

## INTRODUCTION

Making biomedical data more findable, accessible, interoperable, and reusable (the FAIR principles) (Wilkinson et al., 2016) promises to improve how laboratory experiments are performed and interpreted. Adoption of FAIR approaches also responds to concerns from industrial and academic groups about the reproducibility and utility of biomedical research (Arrowsmith, 2011; Baker, 2016; Begley and Ellis, 2012; Prinz et al., 2011) and the adequacy of data-reporting standards (Errington et al., 2014; Morrison, 2014). Several efforts have been launched to repeat published work (https://f1000research.com/channels/PRR), most prominently the Science Exchange Reproducibility Initiative (http://validation.scienceexchange.com/#/reproducibility-initiative). The results of such reproducibility experiments have themselves been controversial (eLife Editorial, 2017; Ioannidis, 2017; Nature Editorial, 2017; Nosek and Errington, 2017.

Rather than focus on a specific published result, the current paper investigates the reproducibility of a prototypical class of cell-based experiments. The research was made possible by the NIH Library of Network-Based Cellular Signatures Program (LINCS) (http://www.lincsproject.org/) and is consistent with its overall goals: generating datasets that describe the responses of cells to perturbation by small-molecule drugs, components of the microenvironment, and gene depletion or overexpression. For such datasets to be broadly useful, they must be reproducible. The experiment analyzed in this paper involves determining how tissue culture cells respond to small-molecule anti-cancer drugs across a dose range. Such experiments compare pre- and post-treatment cell states and require selection of cell types, assay formats, and time frames; they are therefore prototypical of perturbational biological experiments in general. Drug-response assays are widely used in preclinical pharmacology (Cravatt and Gottesfeld, 2010; Schenone et al., 2013) and in the study of cellular pathways (Barretina et al., 2012; Garnett et al., 2012; Heiser et al., 2012).

Cultured cells are typically exposed to anti-cancer drugs or drug-like compounds for several days (commonly three) and the number of viable cells is then determined, either by direct counting using a microscope or by performing a surrogate assay such as CellTiter-Glo (Promega), which measures ATP levels in a cell lysate. With some important caveats, viable cell number is proportional to the amount of ATP in a lysate prepared from those cells (Tolliday, 2010). Several large-scale datasets describing the responses of hundreds of cell lines to libraries of anti-cancer drugs have recently been published (Barretina et al., 2012; Garnett et al., 2012; Haverty et al., 2016; Seashore-Ludlow et al., 2015), but their reproducibility and utility have been debated (Bouhaddou et al. 2016; CCLE Consortium et al., 2015; Haibe-Kains et al. 2013).

**Figure 1.**
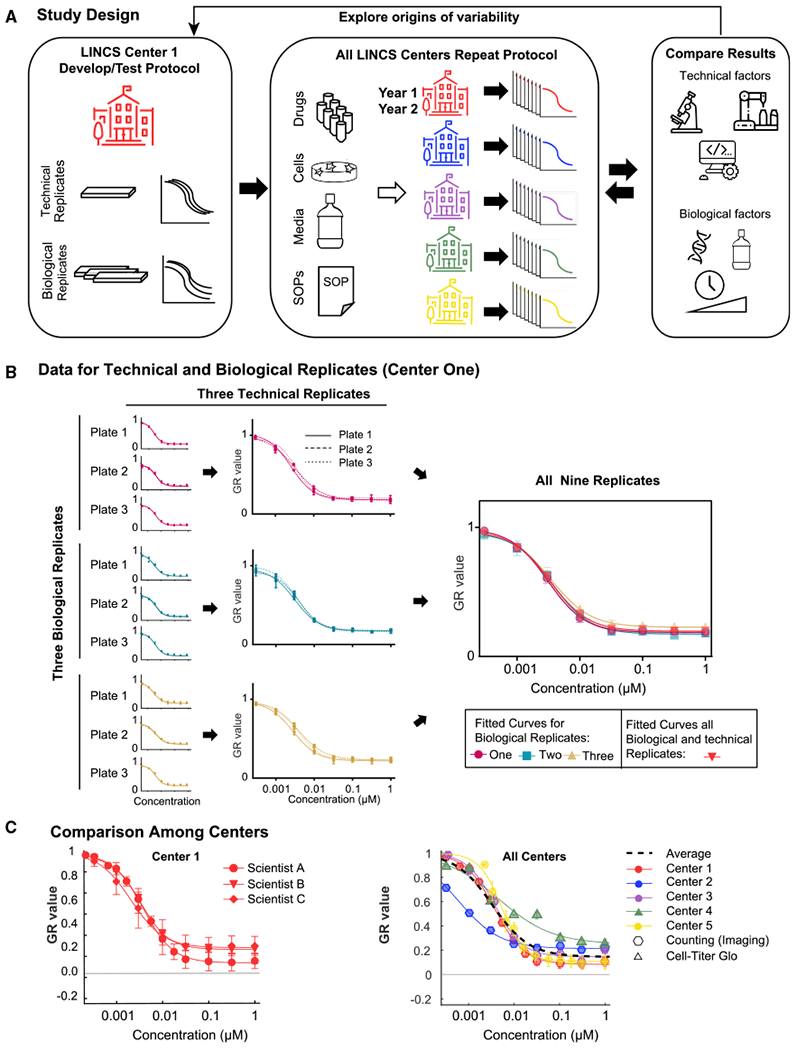
Overview of Workflow (A) Centerone defined the experimental protocol and established within-center reproducibility by assessment of technical (different wells, plates, same day) and biological (different days) replicates. Common stocks of drugs, cells, and media, as well as a standard experimental protocol, were distributed to each of the five data-generation centers. Center one explored the various technical and biological drivers of variability. This information was fed back to the other centers to refine their dose-response measurements. (B) Dose-response curves of MCF 10A treated with the MEK½ inhibitor Trametinib from a typical experiment showing technical and biological replicates. Technical replicates at the well (triplicate wells per plate) and plate (triplicate plates per experiment) levels make up biological replicates (repeats collected on different days in the same laboratory). The red triangles represent the average of the three biological replicates shown. Error bars represent SD of the mean. (C) Independent experiments performed in center one, and in all centers (averages of two or more biological replicates). Circles represent the original dataset, triangles represent data collected by a new technician 2 years after the initial data collection [data shown in (B)], and diamonds represent independently collected data in center one. Inter-center replicates (averages of one or more biological replicates) performed independently at each center. Error bars represent the standard deviation of the mean. See also Figure S1.

Five experimentally focused LINCS Data and Signature Generation centers (DSGCs) measured the sensitivity of the widely used, non-transformed MCF 10A mammary epithelial cell line to eight small-molecule drugs having different protein targets and mechanisms of action. One DSGC (hereafter “center one”) was charged with studying possible sources of irreproducibility identified by inter-center comparison. Investigators in center one had previously shown that conventional drug response measures such as IC_50_ are confounded by variability in rates of cell proliferation arising from variation in plating density, fluctuation in media composition, and intrinsic differences in cell division times (Hafner et al., 2016, 2017a). We corrected for these and other known confounders using the growth rate inhibition (GR) method (Hafner et al., 2016, 2017b; Niepel et al., 2017), thereby focusing the current study on sources of irreproducibility that remain poorly understood. Individual centers were provided with identical aliquots of MCF 10A cells, drugs, and media supplements, as well as a detailed experimental protocol and data analysis procedures (Figure 1A), Supplemental Experimental Procedures). Some variation in the implementation of the protocol was inevitable because not all laboratories had access to the same instruments or the same level of technical expertise; in our view, this is a positive feature of the study because it more fully replicates “real-world” conditions.

In initial experiments, we observed center-to-center variation in GR_50_ measurements of up to 200-fold. Systematic studies revealed factors most likely to be responsible for this variation. In contrast to several recent studies emphasizing genetic instability as a source of variability in sensitivity to anti-cancer drugs (Ben-David et al., 2018), genetic drift did not play a significant role in our studies. Instead, irreproducibility arose from a subtle interplay between experimental methods and poorly characterized sources of biological variation and, to a lesser extent, differences in data analysis (image processing) algorithms. Based on these findings, newly trained technical staff without previous exposure to our protocol could obtain results indistinguishable from assays performed 2 years previously by others. Thus, a sustained commitment to characterizing and controlling for variability in perturbation experiments is both necessary and sufficient to obtain reproducible data.

## RESULTS

### Measuring Drug Responses in Collaboration

To establish the single-center precision of dose-response assays, center one performed technical and biological replicate measurements using MCF 10A cells and the MEK1/2 kinase inhibitor Trametinib at eight concentrations between 0.33 nM and 1 μM (Figures 1B and 1C). For technical replicates, multiple drug dilution series were assayed on one or more microtiter plates on the same day. For biological replicates, three sets of assays were performed, separated by a minimum of one cell passage; each biological replicate involved three technical replicates. In all cases, viable cell number was determined by differentially staining live and dead cells, collecting fluorescence images from each well, segmenting images using software, and then counting all viable cells in all wells (Hafner et al., 2016; Niepel et al., 2017). Sigmoidal curves were fitted to the data and four response metrics derived: potency (GR_50_), maximal efficacy (GR_max_), slope of the dose response curve (Hill Coefficient or h_GR_), and the integrated area over this curve (GR_AOC_). Fitting procedures and response metrics have been described in detail previously (Hafner et al., 2016, 2017b) (Figure S1A), and all routines and data can be accessed on-line or via download at http://www.grcalculator.org/.

We found that response curves for technical replicates were very similar (Figure 1B), showing that purely procedural error resulting from inaccurate pipetting, non-uniform plating, errors in cell counting, etc., were small. Variability in biological replicates as measured by drug potency (log_10_(GR_50_) values) and efficacy (GR_max_ values) was within 1.4 standard deviations for center one (Figure S2) across three different laboratory scientists.

To measure reproducibility across laboratories, while controlling for variation in reagent and genotype, a single center distributed to all other centers identical MCF 10A aliquots, drug stocks, and media additives, as well as a detailed experimental protocol optimized for the cell line-drug pairs under study. This protocol included optimal plating densities, dose-ranges and separation between doses for reliable curve fitting. When individual centers first performed these assays, up to 200-fold variability in GR_50_ values was observed (Figure S3). Differences of this magnitude have previously been observed for large-scale dose-response studies performed by different research teams (Haibe-Kains et al., 2013). To understand the origins of the observed irreproducibility we performed directed and controlled experiments in center one.

### Technical Drivers of Variability

**Figure 2.**
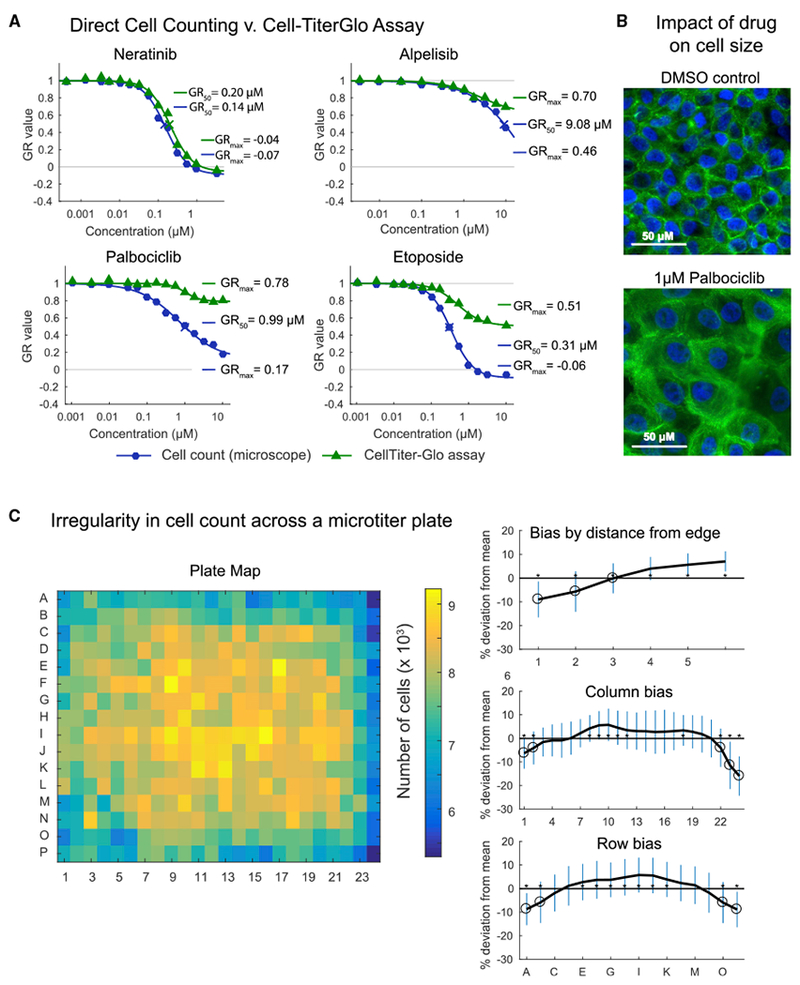
Experimental Causes of Variability (A) Dose-response curves of MCF 10A cells treated with four different drugs measured by image-based cell count or ATP content (CellTiter-Glo) on the same day by center one, which is equivalent to technical replicates. Note the GR_50_ value for alpelisib as measured by CellTiterGlo was not defined. (B) Representative images of MCF 10A cells treated with vehicle control (DMSO) or 1 μM Palbociclib. Cells were stained with Hoechst and phalloidin. Images have been contrast adjusted. (C) Uneven growth of MCF 10A cells in a 384-well plate over the course of 3 days that demonstrates the presence of edge effects. In the heatmap, color represents the number of cells per well, as assessed by imaging. Plots show deviation from mean number (for the full plate based on the distance from the edge, by column, or by row). Error bars represent the standard deviation. Asterisks indicate the row or column differs significantly from all others. See also Figure S2.

First, we studied the origins of the large inter-center variability in estimation of *GR*_*max*_ for the topoisomerase inhibitor Etoposide and CDK4/6 inhibitor Palbociclib. We ascertained that one center had used the CellTiter-Glo ATP-based assay and a luminescence plate reader as a proxy for counting the number of viable cells in a microscope. CellTiter-Glo is among the most commonly used assays for measuring cell viability and was therefore a logical substitute for direct cell counting. However, when we performed side-by-side experiments we found that dose-response curves and GR metrics computed from image-based direct cell counts and CellTiter-Glo were not the same: GR_max_ values (which are unit-less and range from −1 to 1) for the topoisomerase inhibitor Etoposide and CDK4/6 inhibitor Palbociclib differed by 0.61 and 0.57, respectively, for the two assays (GR_50_ values could not be determined for CellTiter-Glo data because GR > 0.5 under all conditions tested (Figure 2A). In contrast, in the case of the EGFR inhibitor Neratinib and the PI3K inhibitor Alpelisib, the differences were smaller, varying by 0.03 and 0.24, respectively. This finding likely explains some of the inter-center differences observed in drug response metrics (Figure S3).

It is known that CellTiter-Glo and direct cell counts are poorly correlated when drugs cause large changes in cell size or alter ATP metabolism, thereby changing the relationship between ATP level in a cell extract and viable cell number (Figure 2B for Palbociclib) (Harris et al., 2016a; Salani et al., 2013; Soliman et al., 2016). The magnitude of this effect depends on the drug being assayed and also on the cell line (Niepel et al., 2017); as a consequence, direct cell counting and CellTiter-Glo can be substituted for each other in some cases but not in others. Thus, a change in protocol justified by pilot studies on a limited number of cell lines and drugs can be problematic when the number or chemical diversity of drugs is increased. In this context, we note that counting viable cells by microscopy is both more direct and cheaper as a measure of viability than ATP levels; CellTiter-Glo is used in place of counting primarily because it is perceived as being easier to perform. The problem is not with CellTiter-Glo itself, which can be reproducible when correctly calibrated, but with equating reduced ATP levels with reduced cell number. Situations in which ATP levels fall in viable or dividing cells might be of interest biologically but identifying these situations requires performing CellTiter-Glo and cell counting assays in parallel.

Edge effects and non-uniform cell growth are a second substantial source of variation in cell based studies performed in microtiter plates (Bushway et al., 2010; Coyle et al., 1989) arising from temperature gradients and uneven evaporation of media. We have observed a variety of irregularities in plating and cell growth that often depend on the batch of microtiter plates, even when plates are obtained from a single highly regarded vendor (Niepel et al., 2017). A variety of approaches are available to minimize edge effects (e.g., placing plates in humidified chambers to reduce evaporation from edge wells), but we find that variation in growth is often confined to specific regions of a plate (Figure 2C) causing systematic errors in dose-response data. Thus, randomized compound dispensing is a valuable way to reduce biases introduced by edge effects and irregular growth. Using an automated liquid-handling robot such as the HP D300e Digital Dispenser, it is possible to dispense compounds directly into microtiter plates in an arbitrary pattern, randomizing the locations of control and technical replicates and converting systematic error into random error, which is more easily modeled (Niepel et al., 2017). The use of washing and dispensing robots also reduces errors that humans make during repetitive pipetting operations; these robots are small, robust, and relatively inexpensive, and their use improves the reproducibility of many medium- and high-throughput cell-based and biochemical studies.

**Figure 3.**
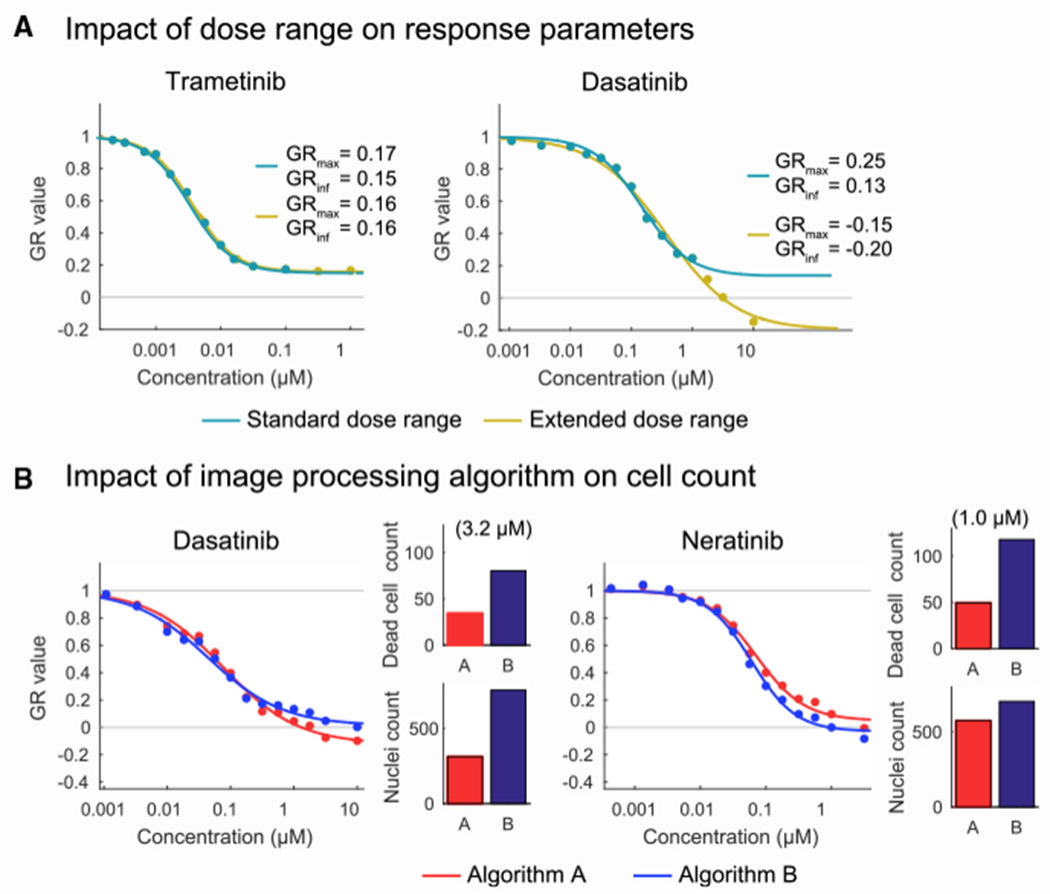
Technical Causes of Variability (A) Dose-response curves of MCF 10A cells treated with Trametinib or Dasatinib fitted to either the extended dose range (up to 1 μM and 10 μM, respectively) or omitting the last order of magnitude. (B) Results of cell counting for MCF 10A cells treated with Dasatinib or Neratinib using two different image processing algorithms (denoted as A (red) and B (blue)) included in the Columbus image analysis software package. (C) Number of dead cells (LIVE/DEAD™ Fixable Red Dead Cell Stain positive) and nuclei (Hoechst positive) counted for MCF 10A cells treated with 3.16 μM Dasatinib or 1 μM Neratinib based on the two different algorithms (corresponding to the plots in C). See also Figures S3 and S4.

A third variable we explored involves the concentration range over which a drug is assayed and the impact of this range on curve fitting and parameter estimation. For example, when Trametinib, a MEK kinase inhibitor, was assayed over a thousand-fold concentration range, growth of MCF 10A cells was fully arrested at ~30 nM (Figure 3A, left plot): phenotypic response did not change even when the dose was increased 100-fold to 1 μM and thus, increasing the dose range had no effect on curve fitting (Figure 3A, left plot). However, when Dasatinib, a poly-selective SRC-family kinase inhibition, was assayed over a thousand-fold range, curve fitting identified a plateau in GR value between 0.3 to 1 μM, but when the dose range was extended to higher drug concentrations GR values became negative (Figure 3A, right plot). Thus, a dose range that is adequate for analysis of Trametinib is not adequate for Dasatinib. This sort of variation is difficult to identify in a high-throughput experiment and suggests that pilot studies are needed to optimize dose ranges for specific compounds. Such variation did not impact reproducibility in our inter-center study because all centers used the identical dose series, but dose range did affect the accuracy of GR_max_ estimation in general.

A fourth source of inter-center variation was apparent among centers that used imaging-based cell counting, particularly when assaying Dasatinib and Neratinib (Figure 3B). Above 1 μM, GR values were reproducibly negative at center one for both drugs but in one other center, GR_max_ was consistently above 0. Follow-up studies showed that the discrepancy arose from the use of image processing algorithms that included dead cells in the “viable cell count” and from over-counting the number of cells when multi-nucleation occurred (Orth et al., 2011; Röyttä et al., 1987). Differences in drug response GR values could be recapitulated in a single laboratory by using different image processing routines and were also evident by visual inspection of the segmented images (Figure 3B). In retrospect, all centers should have processed images in the same way using Dockerized software (List, 2017), but the necessary routines are often built into manufacturer’s proprietary software, making standardization of image analysis dependent on the availability of primary data. This demonstrates the impact of a relatively subtle interplay between biological and technical sources of variability and the importance of locking down all steps in the data processing pipeline from raw measurements to final parameter estimation.

### Biological Factors Impacting Repeatability

Variables that change the biology of drug response, such as media composition, incubation conditions, microenvironment, media volume, and cell density, have been discussed elsewhere (Hafner et al., 2016; Haverty et al., 2016) and were controlled to the greatest extent possible in this study through standardization of reagents and the use of GR metrics. In a truly independent set of assays, experimental variables such as these would need to be considered as additional confounders because it is difficult to fully standardize a reagent as complex as tissue culture media. However, one center performed a preliminary comparison of batches of horse serum, hydrocortisone, cholera toxin, and insulin and found that the effects on drug response were smaller than the sources of variation discussed above.

At the outset of the study, we had anticipated that the origin of the MCF 10A isolate would be an important determinant of drug response. MCF 10A cells have been grown for many years, and karyotyping reveals differences among isolates (Cowell et al., 2005; Kim et al., 2008; Marella et al., 2009; Soule et al., 1990). To investigate the potential impact of genetic drift, we assembled MCF 10A isolates from different laboratories and compared them to each other and to a histone H2B-mCherry-tagged subclone of one of the isolates (Figure S4A); we also examined four subclones from the LINCS MCF 10A master stock. Variation in measured drug response across all isolates and subclones was smaller than what was observed when a single isolate was assayed at different centers. Because highly variable growth rates are a sign of poor technique, we checked doubling times across centers and found them to be similar (Figure S4B). Thus, even though clonal variation can have a substantial effect on drug response and other properties of cultured cells (Ramirez et al., 2016), such variation was not a significant contributor to variability in this study.

**Figure 4.**
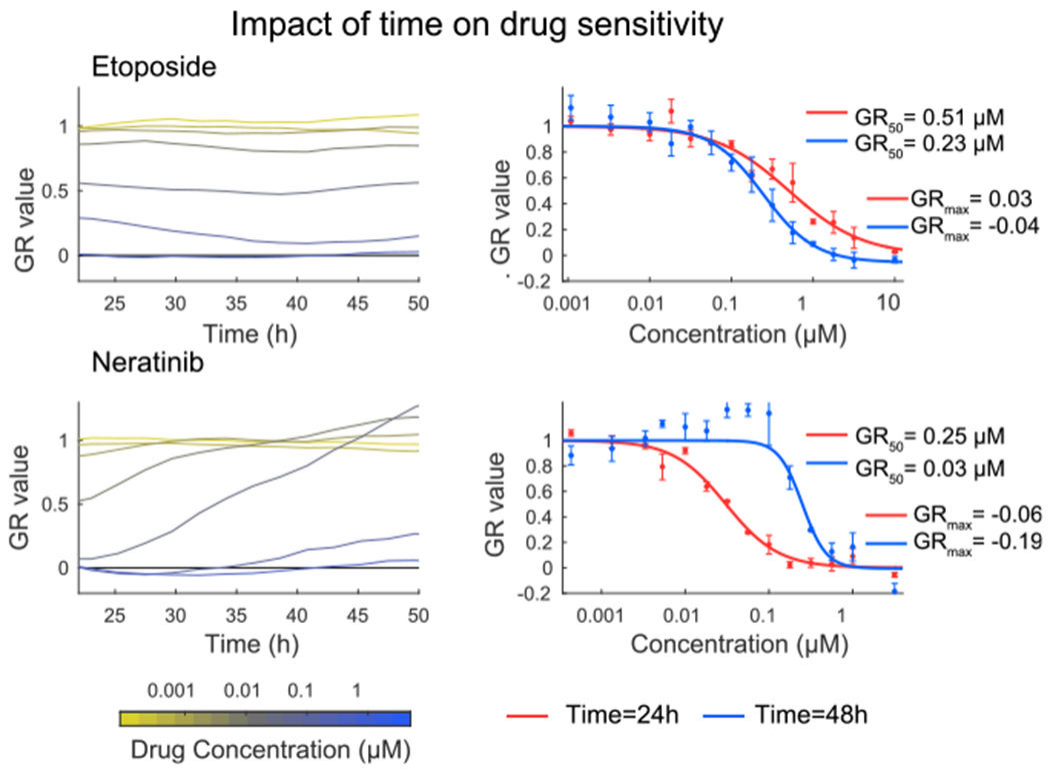
Changes in Drug Response Related to the Underlying Biology Left: Inhibition of MCF 10A growth (12-h instantaneous GR values) measured in a time-lapse, live-cell experiment involving treatment with multiple doses of Etoposide (top) or Neratinib (bottom). Different colors indicate different drug concentrations ranging from 1 nM (yellow) to 10 μM (blue). Right: Dose-response curves derived from 12-h GR values computed at 24 (red) and 48 h (blue) across three biological repeats. Etoposide displays only modest time-dependent effects (top) while neratinib appears to be more effective at inhibiting growth at early time points as compared to later time points (bottom). Error bars, SD. See also Figure S5.

To assay the impact of the time of drug exposure on GR values we performed a live-cell experiment in which cell number was measured every 2 h using an automated high-throughput microscope. When we quantified time-dependent GR values over a 12-h moving window we found a substantial effect in some cases but not others. For example, GR values for cells exposed to Etoposide were nearly constant across all doses throughout a 50-h assay period (Figure 4, top left plot), whereas GR values for Neratinib varied from 0 to 1 over the same period (Figures 4, bottom left plot, and S5), with the highest variability at intermediate drug doses. The temporal dependence of drug response is likely to reflect biological adaptation, drug export, and other factors important in drug mechanism of action (Fallahi-Sichani et al., 2017; Fletcher et al., 2010; Hafner et al., 2016; Harris et al., 2016b; Muranen et al., 2012). These factors remain largely unexplored and are likely to contribute to variation in GR values when protocols are not carefully followed.

### Final Results

To assess success in identifying and controlling for sources of variability in the measurement of drug-dose response, we performed two sets of tests. First, all measurements were repeated in center one 2 years after the first round of studies by an experienced research scientist (Scientist A from the original study, Figures 1 and S2) and by a newly recruited technical associate (Scientist B) who did not have prior experience with drug-response assays. Data were collected in biological triplicate with each replicate separated by a minimum of one cell passage from the next; each biological replicate was assayed in technical triplicate, as described in Figure 1B. Plates, media, supplements, and serum were all from different batches as compared to the original experiments and cells were recovered from independent frozen stocks. However, the protocol remained the same over the 2-year period and involved the same automated compound dispensing and plate washing procedures.

**Figure 5.**
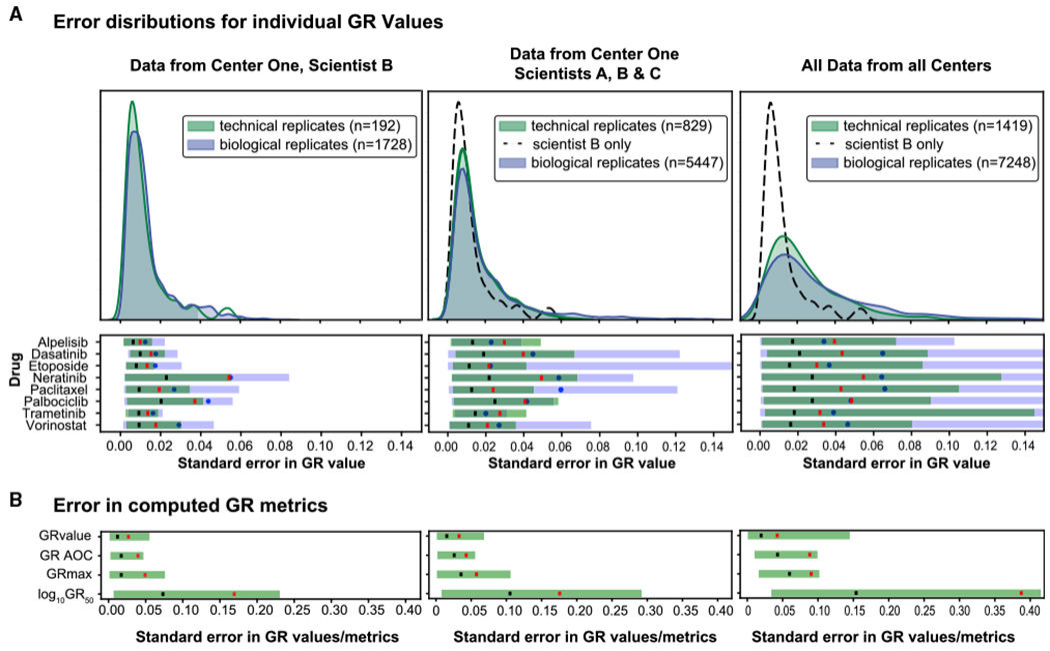
Technical and Biological Variability in Estimating GR Values and Metrics (A) The kernel density estimate (KDE) of the standard error (SE) for measurement of GR values across technical (green curve) or biological (blue curve) replicates for all drugs and doses. The left panel depicts data from center one, scientist B (performed in 2018); the middle panel shows four sets of measurements from all scientists in center one (performed between 2016–2018); and the right panel all data from all centers. The distribution of technical error for Scientist B is duplicated in the middle and right panels as a black dotted line to facilitate comparison. Data for these distributions were derived from GR values for each dose and replicate, not GR metrics obtained from curve fitting. The number of GR value data points used to compute SE is detailed in STAR Methods. The number of SE data points that constitute each KDE is shown in the legend; for the left panel this is 192 SE data points (8 doses × 8 drugs × 3 biological repeats). The lower section of each panel depicts the error in GR value measurements across technical replicates (green) and biological replicates (blue) for each individual drug. (B) The range of SE in GR values compared to the SE in corresponding GR metrics (GR_max_, area over the GR curve (GR AOC), and log_10_GR_50_) for all drugs. The black vertical line (A, lower plots, and B) is the mean technical error for a given drug and the red vertical line demarcates the 90^th^ percentile error across technical replicates (meaning that the error for 90% of GR values or GR metrics is below that value); a blue circle demarcates 90^th^ percentile error across biological replicates.

Data from newly trained Scientist B exhibited similar standard error for biological and technical repeats with a mean standard error for estimation of GR values of 0.012 across all drugs, doses, and repeats. The distribution was long tailed, an apparent consequence of systematic error in assays involving Neratinib (Figure 5A, lower). As shown in Figure 4, GR values for Neratinib are strongly time-dependent and we might therefore expect data for this drug to be sensitive to small variations in procedure. The observed error in GR values corresponds to a difference in the estimation of GR_50_ values of 1.17-fold (mean standard error, which corresponds to a variation of ± 0.07 in log_10_(GR_50_)) while the standard error for 90% of GR_50_ values corresponded to a difference of ~1.5-fold (± 0.18 in log_10_(GR_50_)) (Figure 5B). For all measurements obtained in center one over a period of 2 years, the mean standard error in GR values was 0.015, which is only slightly higher than the error from Scientist B alone. The standard deviations in log_10_(GR_50_) and GR_max_ values obtained by Scientist A over a 2-year period were indistinguishable from each other and there was no observable batch effect for any drug (Figure S2). These distributions represent our best estimate of the error associated with measuring drug-dose response using a single protocol and experimental setup but different consumables; this estimate can therefore be incorporated into future error models. In our opinion these values also represent a good level of accuracy and reproducibility.

**Figure 6.**
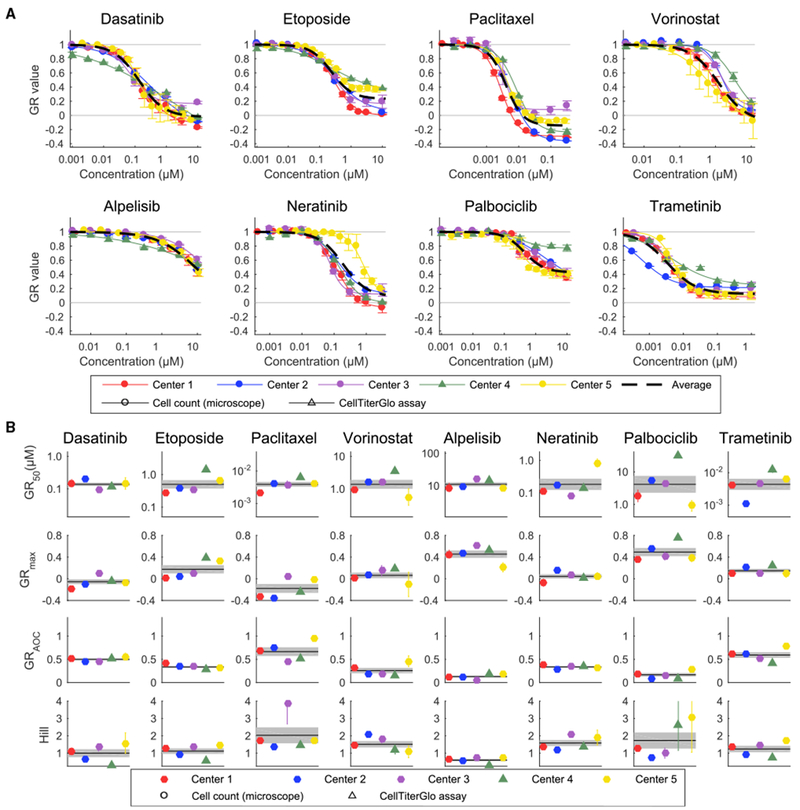
Variability of the Response Measures across Centers (A) Dose-response curves of MCF10A cells treated with eight drugs measured independently by the five centers (circles represent data from image-based assays and triangles from CellTiter-Glo assays). See Figure S6 for underlying replicates. Dotted black lines show the dose-response curve when all independent replicates were averaged. Error bars represent SD of the mean. (B) GR metrics describing the sensitivity of MCF 10A cells to eight drugs measured independently by five centers (circles represent data from image-based assays and triangles from CellTiter-Glo assays). The black line shows the mean sensitivity across all centers, and the gray area shows the standard error of the mean computed from the average of each center. For GR_50_ and GR_max_, error bars represent the standard deviation of the log_10_(GR) values. Note that some data are shared between Figures 6 and S3.

As a second test, centers repeated drug-dose response measurements using their closest approximation to the standard protocol. One center used CellTiter-Glo rather than direct cell counting to estimate viable cell number. Use of this method resulted in greater deviation from the results in center one, as expected from the studies shown in Figures 2, 3, 4, 5, and 6 (e.g., technical error in the CellTiter-Glo data from center four exceeded that of all other centers). Despite such differences in procedure inter-center variability at the end of the study was lower than at the outset, with a standard error in GR value measurement ~2-fold higher than in center one and errors in the estimation of GR_50_ of ~2 standard deviations. The mean standard error for log_10_(GR_50_) across all drugs was ±0.15 while the standard error for 90% of measured GR_50_ values was within ~2.5-fold (± 0.38 in log_10_(GR_50_)) (Figure 5B).

The distribution of standard errors in GR values is long tailed. Although the mean standard errors for technical and biological replicates are comparable, error associated with biological replicates has a longer tail, as illustrated by the cases where the upper 10^th^ to 5^th^ percentile error across biological replicates was greater than the error in technical replicates (Figure 5A, lower panels). For example, center four had consistently high technical variability and low biological variability, possibly a result of their use of the CTG assay. Overall, the largest identifiable source of error in the final data arose from use of the CTG assay as opposed to direct cell counting (Figure 6).

From these data we conclude that it is possible for previously inexperienced individuals to measure drug-dose response with high reliability over an extended period of time and that multiple centers can approximate this level of reproducibility. However, deviations from an SOP (see Supplemental Experimental Procedures) with respect to automation and type of assay, which might be necessary for practical reasons, can have a substantial negative impact.

## DISCUSSION

The observation that a large fraction of biomedical research cannot be reproduced is troubling; it handicaps academic and industrial researchers alike and has generated extensive comment in the scientific and popular press (Arrowsmith, 2011; Baker, 2016; Begley and Ellis, 2012; Prinz et al., 2011; Wilkinson et al., 2016). The key question is why such irreproducibility arises and how it can be overcome; in the absence of systematic studies such as ours, FAIR data will remain little more than an aspiration. In this study, we investigated the precision and reproducibility of a prototypical perturbational experiment performed in cell lines: drug dose-response as measured by cell viability. Perturbational experiments are foundational in genetics, chemical biology, and biochemistry, and when they involve human therapeutics, they are also of translational value. A consortium of five geographically dispersed NIH LINCS centers initially encountered high levels of inter-center variability in estimating drug-potency, even when a common set of reagents was used. Subsequent study in a single center uncovered possible sources of measurement error, resulting in a substantial increase in inter-center reproducibility. Nonetheless, the final level of inter-center variability exceeded what could be achieved in a single laboratory over a period of 2 years by three scientists. We ascribe the remaining irreproducibility to differences in compound handling, pipetting, and cell counting that were not harmonized because of the expense of acquiring the necessary instrumentation and a belief—belied by the final analysis—that counting cells is such a simple procedure that different assays can be substituted for each other without consequence. We believe the final level of intra- and inter-center precision we achieved exceeds the norm for this class of experiments in the current literature (although this is not easy to prove) and that our findings therefore provide a roadmap for future studies of reproducibility in other settings.

At the outset of the study we had hoped that comparison of data across centers would serve to identify the specific biological, experimental, and computational factors that had the largest impact on data reproducibility. However, we discovered that most examples of irreproducibility are themselves irreproducible and that technical factors responsible for any specific outlier measurement are difficult to pin down. We therefore undertook a systematic study of the assay itself, in a single center, with an eye to identifying those variables with the greatest impact on reproducibility. We found that these variables differed from what we expected a priori. For example, isolate-to-isolate differences in MCF 10A cultures had less of an effect on drug response assays (Figure S4A) than the ways in which drugs and cells were plated into multi-well plates and counted (Figures 2 and 3).

In general, we found that irreproducibility most commonly arose from unexpected interplay between experimental protocol and true biological variability. For example, estimating cell number from ATP levels using the CellTiter-Glo assay produces very similar results to direct cell counting with a microscope in the case of Neratinib, but this is not true for Etoposide or Palbociclib (Figure 2A). The discrepancy most likely arises because ATP levels in lysates of drug-treated cells vary for reasons other than loss of viability; these include changes in cell size and metabolism. We have previously shown that the density at which cells are assayed can have a dramatic effect on drug response (Hafner et al., 2016), but this too is context dependent. For some cell line-drug pairs, density has little or no effect, whereas for other pairs it increases drug sensitivity and for yet others it has the opposite effect. This observation has important implications for the design of experiments in which diverse compounds are screened: pilot studies on a limited range of conditions (dose and drug identity in this work) cannot necessarily be extrapolated to large datasets and are not a sound basis for substituting indirect assays for direct assays. The tendency for even experienced investigators to substitute assays for each other, or to implement historical methods rather than standardized protocols (SOPs), is undoubtedly a source of irreproducibility.

Several lines of evidence suggest that context dependence in drug response reflects true changes in the underlying biology and not flaws in assay methodology itself. For example, cell density directly impacts media conditioning and the strength of autocrine signaling, which in turn changes responsiveness to some drugs but not others (Wilson et al., 2012; Yonesaka et al., 2008). Thus, even in cell lines, drug response is not a simple biological process, and it is easy to envision ways in which changes in measurement procedure that might have no effect in one cell type or biological setting could affect results obtained in other settings. At the current state of knowledge, there is no substitute for empirical studies that carefully assess the range of conditions over which data remain reliable and precise for cell lines and drugs of interest. Moreover, the most direct assay—not a convenient substitute—should be used to score a phenotype whenever possible. Unfortunately, when the goal is collection of a large dataset, a prerequisite for most machine-learning approaches, attention to biological factors known to be important from conventional cell biology studies is often de-emphasized in favor of throughput.

Data-processing routines are important for reproducibility (Sandve et al., 2013). Data and data-analysis routines can interact in multiple ways, some of which are clear in retrospect but not necessarily anticipated. For example, collecting eight-point dose-response curves generally represents good practice, but it is essential that the dose range effectively span the GEC_50_ (the mid-point of the response). When this is not the case (as illustrated by Figure 3A), curve fitting is underdetermined and response metrics become unreliable. In many cases problems with dose range are not evident until an initial assay has been performed and an iterative approach is therefore necessary. Iteration is straightforward in small-scale studies, but more difficult in large-scale screens; for a large dataset, data-processing routines must be developed to automatically identify and flag problems with dose range. Additionally, accurate reporting of dose range is necessary to provide a bound-to-drug sensitivity measurement. Another example of data-processing challenges involves imaging software for automated cell counting: such routines should be optimized for cells that grow and respond to drugs in different ways (Figure 3B) and must be tested for performance at high and low cell densities.

Processing pipelines for the type of data collected in this study are much less developed than the pipelines commonly used for genomics data (Ashley, 2016; Bao et al., 2014; Lam et al., 2011), but much can be learned from the comparison. For example, computational platforms with provenance such as Galaxy (Goecks et al., 2010), or Sage Bionetworks’ Synapse (Omberg et al., 2013) have been developed to support data sharing, reproducible analyses, and transparent pipelines, with a primary focus on genomics data. Some of these best practices have already been adapted to the analysis of LINCS dose-response data (see STAR Methods). Image-processing algorithms present a unique challenge in that they are frequently embedded in proprietary software linked to a specific data acquisition microscope, which complicates common analysis across laboratories; publicly available image analysis platforms are preferable (Carpenter et al., 2006).

### Elements of a Reproducible Workflow

**Figure 7.**
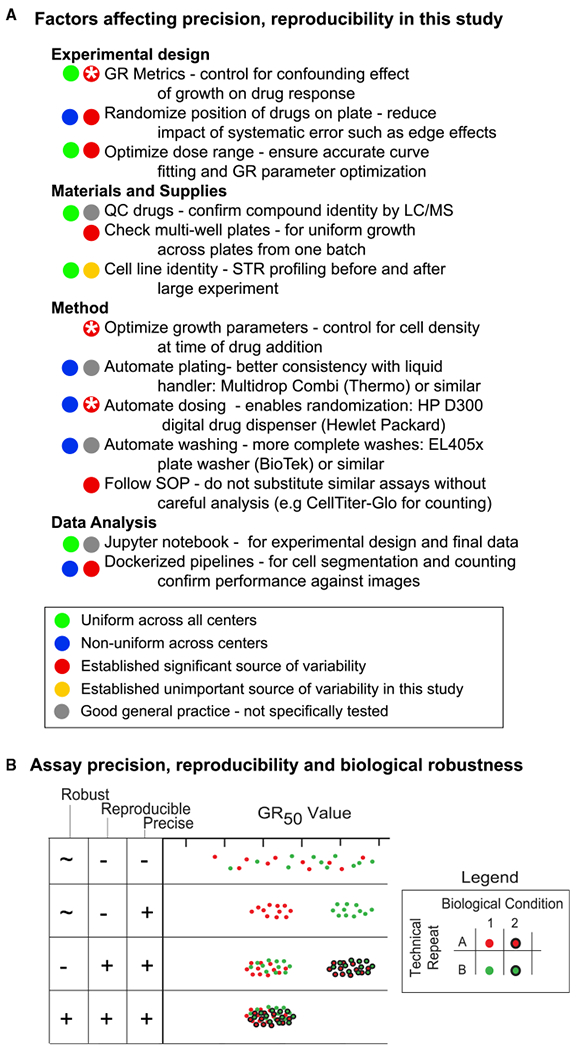
Best Practices for Dose-Response Measurement Experiments (A) Summary of findings in this and related studied with respect to experimental and technical variability in dose response studies at the experimental design, materials, methods, and analysis stages; “*” indicates sources of variability that have been thoroughly investigated in a previous paper (Hafner et al., 2016). (B) Differences between precision, robustness, and reproducibility; see text for details.

The elements of a workflow for reproducible collection of dose-response data are fairly simple conceptually (Figure 7A) although not necessarily easy to implement: (1) standardization of reagents, including obtaining cell lines directly from repositories such as the ATCC, performing mass spectrometry-based quality control of small-molecule drugs, and tracking lot numbers for all media additives; (2) standardized data processing starting with raw data and metadata through to reporting of final results; (3) use of automation to improve reliability and enable experimental designs too complex or labor intensive for humans to execute reliably—in many cases, this involves simple and relatively inexpensive bench-top dispensing and washing—and (4) close attention to metrology (analytical chemistry), measurement technology, and internal quality controls. The first two points are obvious, but not all laboratories are equipped in the same way and some data-processing routines are embedded in a non-obvious way in instrument software. In the current work, a major benefit of automation is that it makes random plate layouts feasible, thereby changing systematic edge effects into random error that has less effect on dose-response curve fitting. In the case of dose-response data, metrology focuses on variability among technical and biological replicates, assessment of edge effects, and outlier detection. Edge effects and other spatial artifacts can be identified by statistical analysis (Mazoure et al., 2017) and plate-wise data visualization (Boutros et al., 2006). Spatial artifacts can then be removed with plate-level normalization such as LOESS/LOWESS smoothing (Boutros et al., 2006; Pelz et al., 2010), spatial autocorrelation (Lachmann et al., 2016), or statistical modeling (Mazoure et al., 2017).

A contribution of the current study is to show that future execution of reproducible drug-dose-response assays in different cell types requires systematic experimentation aimed at establishing the robustness of assays over a full range of biological settings and cell types. Such robustness is distinct from conventional measures of assay performance such as precision or repeatability in a single biological setting (Figure 7B). Testing of this type is not routinely performed for the simple reason that establishing and maintaining robust and reproducible assays is time consuming and expensive: we estimate that reproducibility adds ~20% to the total cost of a large-scale study such as drug-response experiments in panels of cell lines (AlQuraishi and Sorger, 2016). Iterative experimental design is also essential, even though it has been argued that this is not feasible for large-scale studies (Harris, 2017).

### Conclusions

A question raised by our analysis is whether, given their variability and context-dependence, drug response assays performed *in vitro* are useful for understanding drug response in other settings, human patients in particular. Concern about the translatability of *in vitro* experiments is long-standing, but we think the current work provides grounds for optimism rather than additional worry. Simply put, if *in vitro* data cannot be reproduced from one laboratory to the next, then it is no wonder that they cannot easily be reproduced in humans; conversely, paying greater attention to accurate and reproducible *in vitro* data are likely to improve translation. Moreover, many of the factors that appear to represent irreproducibility in fact arise from biologically meaningful variation. These include the time-dependence of drug response, the impact of non-genetic heterogeneity at a single-cell level, and the influence of growth conditions and environmental factors. The simple assays of drug response in current use are unable to correct for such variability, and the problem is made worse by “kit-based science” in which technical validation of assays is left to vendors. However, if the challenge of understanding biological variability at a mechanistic level is embraced, it seems likely that we will improve our ability to conduct *in vitro* assays reproducibly and apply data obtained in cell lines to human patients (Goodspeed et al., 2016). We note that RNAi, CRISPR, and other perturbational experiments in which phenotypes are measured in cell culture are likely to involve many of the same variables as the dose-response experiments studied here.

Despite a push for adherence to the FAIR principles there is currently no consensus that the necessary investment is worthwhile, nor do incentives exist in the publication or funding processes for individual research scientists to meet FAIR standards (AlQuraishi and Sorger, 2016). Data repositories are essential, but we also require better training in metrology, analytical chemistry, and statistical quality control. In developing incentives and training programs, we must also recognize that reproducible research is a public good whose costs are borne by individual investigators and whose benefits are conferred to the community as a whole.

## STAR★METHODS

### LEAD CONTACT AND MATERIALS AVAILABILITY

Further information and requests for resources and reagents should be directed to, and will be fulfilled by, the Lead Contact, Laura Heiser (heiserl@ohsu.edu).

#### Materials Availability Statement

This study did not generate new unique reagents.

### EXPERIMENTAL MODEL AND SUBJECT DETAILS

Three isolates of the non-malignant female human breast epithelial MCF 10A cell line, here referred to as MCF 10A-GM, MCF 10A-OHSU, and MCF 10A-HMS, were sourced independently from the ATCC and then passaged in separate institutions; use of these lines was intended to replicate the common practice of maintaining local cell stocks. MCF 10A-H2B-mCherry cells were created by inserting an H2B-mCherry expression cassette into the AAVS1 safe harbor genomic locus of MCF 10A-HMS using CRISPR/Cas9 (Hafner et al., 2016). All lines were confirmed to be MCF 10A cells by STR profiling (Table S1), and confirmed to have stable karyotypes by g-banding 47,XX,i(1)(q10),+del(1)(q12q32),add(3)(p13),add(8)(p23),add(9)p(14). All lines were cultured in DMEM/F12 base media (Invitrogen #11330-032) supplemented with 5% horse serum, 0.5 μg/mL hydrocortisone, 20 ng/mL rhEGF, 10 μg/mL insulin, 100 ng/mL cholera toxin, and 100 units/mL penicillin and 100 μg/mL streptomycin as described previously (Debnath et al., 2003). Base media, horse serum, hydrocortisone, rhEGF, insulin, and cholera toxin where purchased by the MEP-LINCS Center and distributed to the remaining experimental sites. MCF 10A-GM was expanded by Gordon Mills at MD Anderson Cancer Center and distributed to all experimental sites. Cell identity was confirmed at individual experimental sites by short tandem repeat (STR) profiling, and the cells were found to be free of mycoplasma prior to performing experiments.

### METHOD DETAILS

The experimental and computational protocols to measure drug response are described in detail in two prior publications (Hafner et al., 2017b; Niepel et al., 2017). The following protocol (available in full below) was suggested for this study: cells were plated at 750 cells per well in 60 μL of media in 384-well plates using automated plate fillers and incubated for 24 h prior to drug addition. Drugs were added at the indicated doses with a D300 Digital Dispenser (Hewlett-Packard), and cells were further incubated for 72 h. At the time of drug addition and at the endpoint of the experiment, cells were stained with Hoechst and LIVE/DEAD™ Fixable Red Dead Cell Stain (Thermo Fisher Scientific) and cell numbers were determined by imaging as described (Hafner et al., 2016; Niepel et al., 2017) or by the CellTiter-Glo assay (Promega). Some details of the experimental protocol differed across Centers and overtime, e.g., manually dispensing of drugs or use of 96-well plates. The data included in Figures 1C and S2 (Scientist C) were collected for a separate project using different stocks of consumable reagents, and included here as an additional comparison. In these experiments, cells were treated via pin transfer, and HMS isolate 3 MCF10A cells were used.

For live-cell experiments with MCF 10A-H2B-mCherry, cell counts were performed by imaging plates in an 2 hr interval over the course of 96 hours (only first 50 hours shown) (Hafner et al., 2016; Niepel et al., 2017). Data analysis was performed as described previously (Hafner et al., 2016; Niepel et al., 2017).

The evaluation of irregularities in growth across microtiter plates was performed by plating MCF 10A cells at 750 cells per well in 60 μL of media in 384-well plates using automated plate fillers and determining cell numbers after 96 h through imaging as described (Hafner et al., 2016; Niepel et al., 2017).

Drugs were obtained from commercial vendors by HMS LINCS, tested for identity and purity by LC/MS in house as described in detail in the drug collection section of the HMS LINCS Database (http://lincs.hms.harvard.edu/db/sm/), and distributed as 10 mM stock solutions dissolved in DMSO to all experimental sites. See Key Resources Table for additional metadata.

#### Measuring Drug Responses – SOP

##### General Considerations

The two main considerations in measuring drug responses in cell lines are that the results are reproducible and representative of the relevant underlying biology of the system. To improve reproducibility we point out specific experimental steps that are prone to introducing variability and articulate what steps can be taken to minimize this variability. To ensure that the results are representative of the underlying biology we point out specific experimental conditions that should be optimized for each drug-cell line condition. For example, some drug-cell line interactions change with cell density and/or are dependent on cell state, so it is important to maintain constant plating numbers within an appropriate density range from one experiment to the next. Although always plating cells at high density so there is little or no growth might produce reproducible results that suggest a cell line is resistant to drug, this result would not necessarily be representative of how the drug actually acts on dividing cells. Experimental design therefore must achieve both goals - reproducibility and representativeness.

Automation is one key way to improve reproducibility. In particular when working with 384 well plates any form of manual manipulation will introduce unacceptable levels of variation. Ideally, every step (plating, treatment, measurement, and analysis) should be automated to reduce user-induced artifacts.

##### Step-By-Step Protocol

###### Plating Cells.

1) Grow MCF10A cells following protocol provided by Gray/Mills.
  a) It is important the cells are in mid-log phase and not in a state of arrest or quiescence since they will otherwise need more than 24 hours to become proliferative again.
2) Harvest and count cells following protocol provided by Gray/Mills.
  a) Make sure that during the detaching process all cells get harvested and that the cells do not clump which will make accurate counting and dispensing difficult.
  b) Cells should quickly be brought up in complete growth media and traces of detaching solution should be removed by centrifugation to minimize stress for the cells.
  c) Automated cell counters may not give the most accurate counts, but they will speed up the process when many cell solutions need to be counted and they will improve reproducibility
3) Plate cells at 750 cells/well in 60-ul complete media in four standard 384 well plates compatible with downstream assay of cell number. (ALTERNATIVE – Plate 2250 cells/well in 200ul media if using 96 well plates.)
  a) An accurate count here is not sufficient to estimate the number of cells in the well at the time of drug treatment. There is too much variability in the the number of cells that actually adhere and the time it takes for cells to start growing after plating. We therefore use one plate of the four plates to obtain an accurate pre-treatment cell count.
  a) Especially for sensitive cells it is important to stain for dead cells to ensure that the correct number of live cells gets plated.
  b) Ideally use a fully automated cell dispenser.
  c) Take care to gently resuspend cells if plating takes more than a couple of minutes as cells will settle which will lead to uneven dispensing.
  d) Place plates on a benchtop, sheltered from direct warm or cool air from the heating system, for 20 minutes to allow the cells to settle. Cells may distribute unevenly if they are placed directly in the incubator due to vibration of the shelves.
  e) Move plates to an incubator. If the incubator is opened often, it is advisable to place plates into secondary containment (we use a tupperware container lined with moist paper towels) to reduce temperature and CO2 fluctuations, in particular of the edge wells.
4) Incubate cells for 24 hours.
  a) Cells will show a bit of a lag phase after plating, either due to a slowdown of growth during the expansion of the cells or due to stress induced by plating. It is advisable to observe for a new cell line if cells are actively cycling after plating.
  b) We have observed some synchronization after cell plating as well. Again, this is likely due to a cell cycle arrest present in the cells at the time of plating.

###### Treating Cells.

5) Treat cells in three plates with drugs in a nine-point SQRT(10)-fold dilution series covering four orders of magnitude starting at the highest dose according to the table below using an HP D300 Drug Dispenser.
  a) The time of addition of drug is considered t=0.
  b) The experimental design should be such that the three plates represent a technical triplicate of the overall experiment. Since there is plate-to-plate variation it is best to have the technical repeats on different plates.
  c) Automation is the most important feature. And ideally, we want to minimize the addition of extraneous media. So treatment with a D300 or pin transfer is ideal.
  d) If no D300 drug dispenser is available, prepare the drugs at the right concentration and transfer them in 10 μl into each well using a multi-channel pipette.
6) At time t=0 assay the fourth (untreated control) plate (see below).
7) Incubate the treated cells for up to an additional 72 hours.
  a) Ensure that in the DMSO-treated control wells cells are still dividing actively at the end of the experiment.
  b) Fast growing cell lines can be measured after two days while slower growing lines can be incubated for three or even four days.
8) At time t=3 days assay the technical triplicate plates (see below).

###### Measuring Cell Numbers.

9) At the indicated time points perform your preferred assay to determine the relative cell number for each well.
  a) The preferred method to analyze cell number is to count them by microscopy assays to get a direct count of viable cells (see assay below).
  b) ALTERNATIVE – Proxy assays such as CellTiter-Glo or AlamarBlue will work for the GR calculations, however DNA, ATP, or other proxy-markers may be affected by drug response independent of the actual cell number.
10) Add 20μl of staining solution (1:1000 LIVE/DEAD Far Red Dead Cell Stain (Thermo Fisher Scientific, L-34974), 2 μM Hoechst 33342 (Thermo Fisher Scientific, 62249), 10% OptiPrep (Sigma-Aldrich, D1556-250ML) in PBS). Incubate for 30min at RT. Add 20μl of fixing solution (3% formaldehyde (Sigma Aldrich, F8775-500ML), 20% OptiPrep (Sigma-Aldrich, D1556-250ML) in PBS). Incubate for 30min at RT. Remove 90μl of supernatant and replace with 90μl of PBS and proceed to scanning.
  a) NOTE – Add the staining and fixing solution with an automated pipettor, holding it at an approximately 45 degree angle and touching the side wall of the tube. The solutions should run down the side wall of the well and accumulate at the bottom of the well due to their increasing density.
  b) ALTERNATIVE – Stain and fix cells by adding 20μl of 8 μM Hoechst 33342 (Thermo Fisher Scientific, 62249) in 12% formaldehyde (Sigma Aldrich, F8775-500ML). Incubate for at least 1h before proceeding to scan. This method does not distinguish between live and dead cells directly, even though apoptotic cells should have grossly altered morphology which can be recognized by image analysis software.
11) Scan each treated well of the 384 well plates.
  a) If possible, scan the entire well area to improve count accuracy for low cell numbers or unevenly distributed cells.
12) Use your favorite image analysis algorithms to count live cells.
  a) Use a standard nuclei detection algorithm. Be sure to impose min/max levels for area or brightness to exclude nuclear fragments.
  b) If using the LIVE/DEAD stain, do not count any nuclei that are LIVE/DEAD-positive.

###### Record Results.

13) Record the measured cell numbers or proxy measurements according to the DR2.0 standards.
  a) Data standards are detailed in a separate document.
  b) Be sure to record all necessary pieces of information so the results from different Centers can be aggregated and compared

###### Calculating Drug Sensitivity.

14) Calculate growth-rate inhibition (GR) values for each drug dose and fit the resulting curve with a sigmoid to extract GR_50_, GR_inf_, and GR_hill_.
  a) Calculation of GR metrics are detailed in a separate document.

###### Drug Information.

**Table:** 

Drug	HMSLid	Primary target	Highest dose (uM)	Stock (mM)
Paclitaxel	10102	microtubules	1	10
Alpelisib/BYL719	10233	PI3Ka	10	10
Neratinib/HKI272	10018	EGFR/HER2	3.16	10
Dasatinib	10020	BCR/ABL	10	10
Trametinib/GSK1120212	10142	MEK	1	10
Palbociclib/PD0332991	10071	CDK4/6	3.16	10
Vorinostat	10282	HDAC	10	10
Etoposide	10250	Topoisomerase	10	10

#### QUANTIFICATION AND STATISTICAL ANALYSIS

The technical variability associated with data collected by each Center or scientist in Center one was computed for each drug-dose pair as the standard error (SE; Equation 1) in GR value across all technical replicates per biological replicate. Note that GR values, not GR metrics derived from curve fitting were used for this calculation. The number of data points considered for calculating SE in technical replicates varied by Center/scientist and is shown in the table under the column “# Technical replicates per biological replicate”. The number of SE values per drug-dose pair is equal to the number of biological replicates. For example, the distribution of standard error (technical replicates) for Center 1, Scientist B is made up of 192 SE data points (8 drugs * 8 doses * 3 biological replicates).

Equation 1a $$\mathrm{SE}=\frac{\sigma }{\sqrt{n}}$$

Equation 1b $$\sigma =\sqrt{\frac{\sum {\left(x_{i}-\mu \right)}^{2}}{n-1}}$$

For a given drug-dose pair, σ is the standard deviation computed across GR values, *n* is the number of technical or biological replicates, *x_i_*,-is the GR value measured in a certain replicate *i, μ* is the mean GR value across replicates.

The biological variability of each Center or scientist was also computed for each drug-dose pair as the standard error (SE; Equation 1) in GR value across all biological replicates such that each SE computation was based on data from only one technical replicate per biological replicate. The number of data points used to compute each SE value is equal to the number of biological replicates. The number of SE values per drug-dose pair is equal to ${\left(\begin{array}{c} t\\ 1 \end{array}\right)}^{b}$ or *t*^*b*^ where *b* is the number of biological replicate plates, *t* is the number of technical replicates per biological replicate. For example, the number of SE values (data points) computed per drug-dose pair for Center 1, Scientist B is 3^3^ = 27. The total number of drug-dose pairs in a complete dataset for each Center is 64. Hence, the distribution of standard error for biological replicates associated with data collected in Center 1 by Scientist B is computed from 1728 data points.

**Table:** 

Center/Scientist	# Technical replicates per biological replicate	# Biological replicates
Center 1, Scientist A (2019)	9 (3 wells × 3 plates)	3
Center 1, Scientist A (2017)	2	3
Center 1, Scientist B	9 (3 wells × 3 plates)	3
Center 1, Scientist C	4	2
Center 2	4	1
Center 3	3	2
Center 4	3	2
Center 5	2	2

### DATA AND CODE AVAILABILITY

Analysis of variability in GR values or metrics measured across centers is recorded in Jupyter notebooks. These notebooks document blocks of executable code alongside human-readable descriptions of the methods used to compute variability, and can be re-run by the reader to reproduce the results described. Jupyter notebooks for experimental design and data analysis are available: https://github.com/labsyspharm/MCF10A_DR_reproducibility and https://github.com/datarail/datrail.

The data from each Center and a list of best practices are available at http://www.grcalculator.org/grbrowser/ under ‘LINCS MCF10A Common Project’.

The data from Scientist C are available: http://lincs.hms.harvard.edu/db/datasets/20343/ and http://lincs.hms.harvard.edu/db/datasets/20344/

All data have also been deposited on Synapse: (synapse.org) syn18456348. The final drug response results (mean GR values and GR metrics) generated by all LINCS Centers (Related to Figure 5) are under Synapse: syn18478968. The time course data (Related to Figures 4 and S5) are under Synapse: syn18478971. All technical and biological GR values for each Center (Related to Figures 5 and S6) are under Synapse: syn18475380.

## Supplementary Material

1

## Figures and Tables

**Table:** KEY RESOURCES TABLE

REAGENT or RESOURCE	SOURCE	IDENTIFIER
Chemicals, Peptides, and Recombinant Proteins		
Horse Serum	Sigma-Aldrich	Cat # H1138, Lot # 12B496
Penicillin/Streptomycin	Invitrogen	Cat # 15070-063, Lot # 1697552
Hydrocortisone	Sigma-Aldrich	Cat # H-4001, Lot # SLBN5690V
Epidermal growth factor	R&D Systems	Cat # 236-EG, Lot # HLM7515071
Insulin	Sigma-Aldrich	Cat # I9278, Lot # SLBP1369V
Cholera toxin	Sigma-Aldrich	Cat # C8052, Lot # 095M4093V
Alpelisib	MedChem Express	Cat # HY-15244, Lot # 06192
Dasatinib	MedChem Express	Cat # HY-10191, Lot # 13044
Etoposide	MedChem Express	Cat # HY-13629, Lot # 11793
Neratinib	MedChem Express	Cat # HY-32721, Lot # 10283
Paclitaxel	MedChem Express	Cat # HY-B0015, Lot #18138
Palbociclib	MedChem Express	Cat # HY-50767, Lot # 16349
Trametinib	MedChem Express	Cat # HY-10999, Lot # 07378
Vorinostat	MedChem Express	Cat # HY-10221, Lot # 09386
Deposited Data		
Mean GR values and metrics for all Centers	this paper	Synapse: syn18478968
GR values and metrics for all Centers/Scientists	this paper	Synapse: syn18475380
GR values and metrics for timecourse	this paper	Synapse: syn18478971
Experimental Models: Cell Lines		
MCF10A	ATCC	CRL-10317; RRID CVCL_0598
MCF 10A-H2B-mCherry	Hafner et al. 2016	N/A
Software and Algorithms		
MATLAB (R2016b)	MathWorks	https://mathworks.com/products/matlab.html
Columbus (v2.7.0)	Perkin Elmer, Waltham, MA	http://perkinelmer.com/product/image-data-storage-and-analysis-system-columbus
DataRail	Hafner et al. 2017b	https://github.com/datarail/datarail

## Highlights

- Implementing FAIR data standards requires identification of experimental confounders
- Five labs performed the same experiment on mammalian cells and compared results
- Several factors affecting reproducibility were explored
- Biological context had an unexpected impact on the robustness of cell-based assays
