# Supplementary material for: A Multi-center Study on the Reproducibility of Drug-Response Assays in Mammalian Cell Lines
Source: Cell Syst. Author manuscript; Available in PMC 2020 Jul 24. (PMC6700527; doi:10.1016/j.cels.2019.06.005)
Supplement: 1 [file NIHMS1534612-supplement-1.pdf]

Cell Systems, Volume *9*

Supplemental Information

A Multi-center Study on the Reproducibility of Drug-Response Assays in Mammalian Cell Lines

Mario Niepel, Marc Hafner, Caitlin E. Mills, Kartik Subramanian, Elizabeth H. Williams, Mirra Chung, Benjamin Gaudio, Anne Marie Barrette, Alan D. Stern, Bin Hu, James E. Korkola, LINCS Consortium, Joe W. Gray, Marc R. Birtwistle, Laura M. Heiser, and Peter K. Sorger

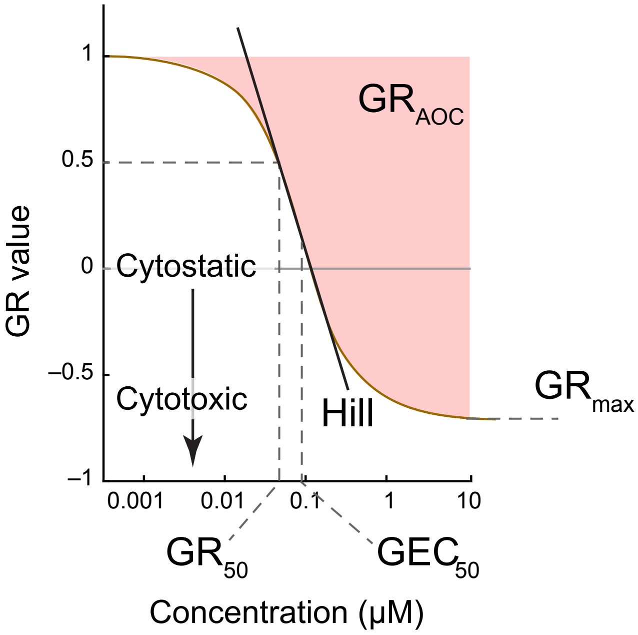

Figure S1, Related to Figure 1

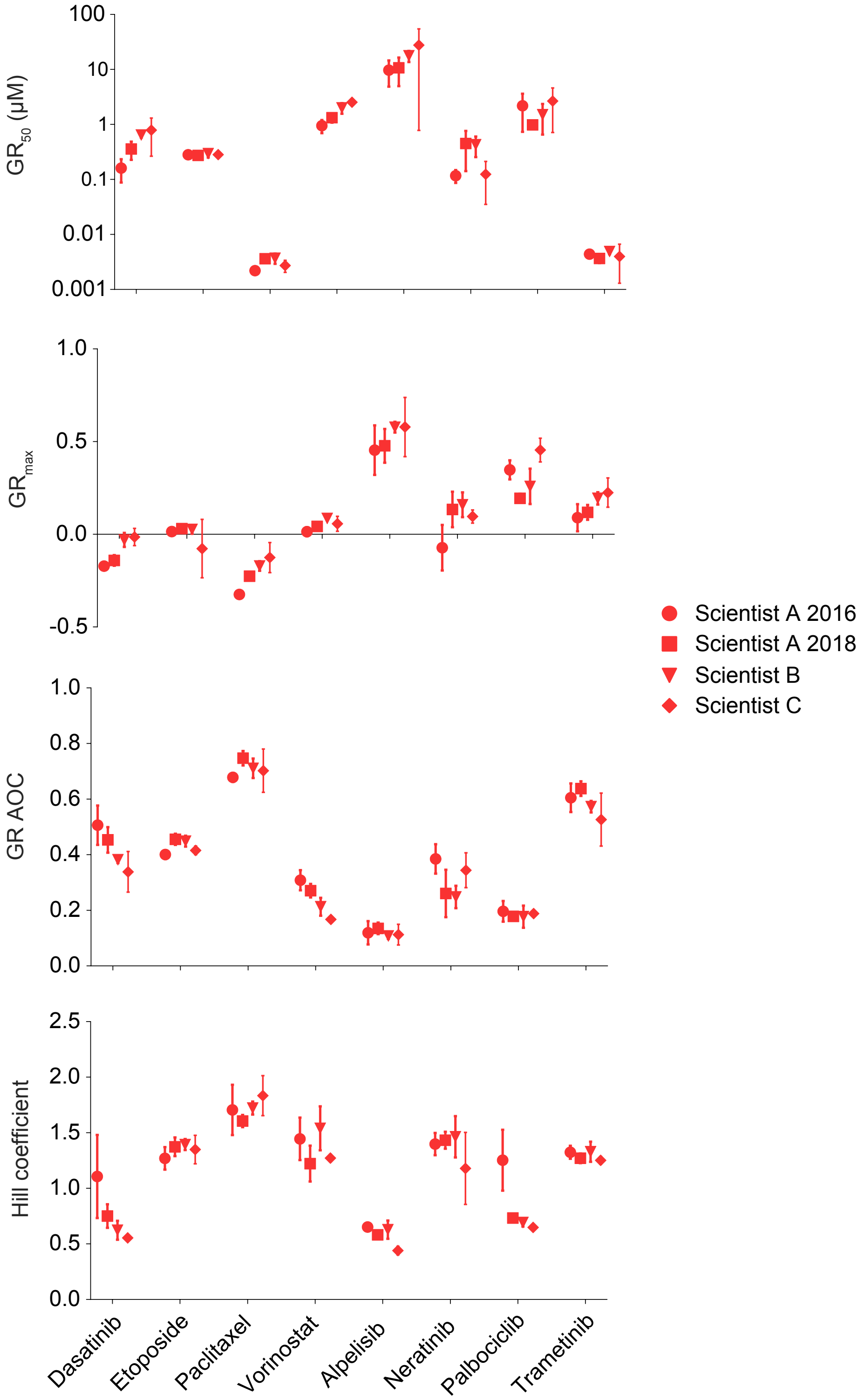

Figure S2, Related to Figure 2

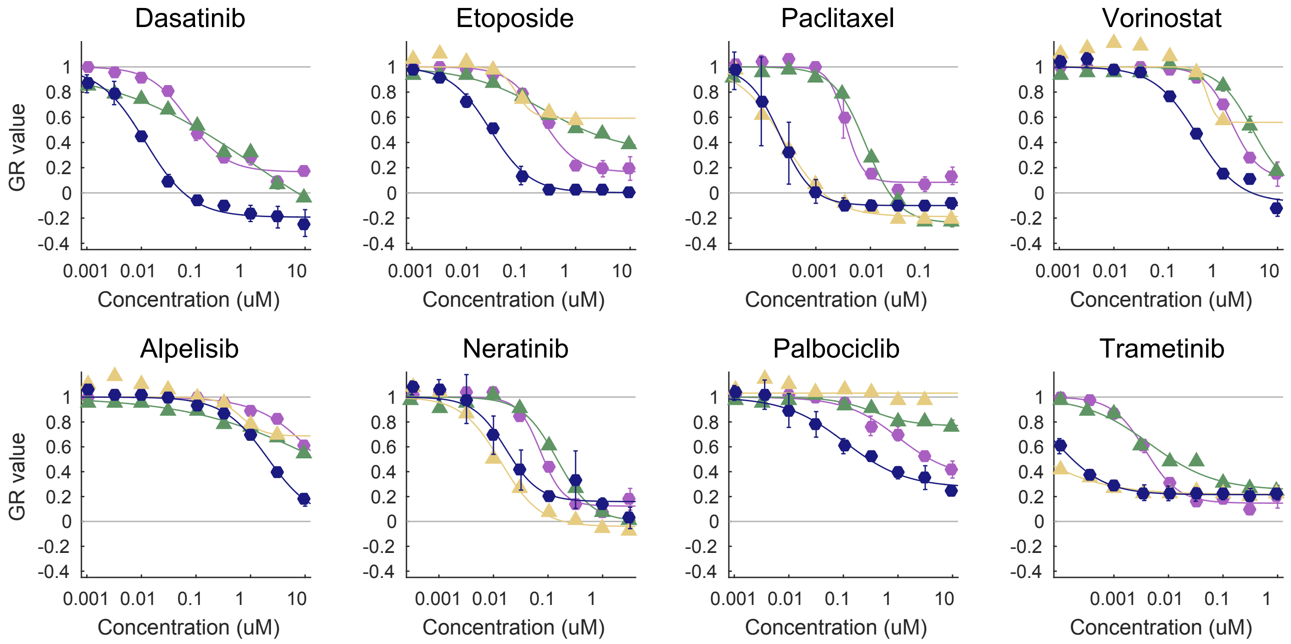

	Drug dilution performed at:	Cell culture performed at:	Readout assay:	
Preliminary 1	Center 3	Center 3	Imaging	
Preliminary 2	Center 3	Center 4	CTG	
Center 3	Center 3	Center 3	Imaging	
Center 4	Center 4	Center 4	CTG	

Figure S3, Related to Figure 3

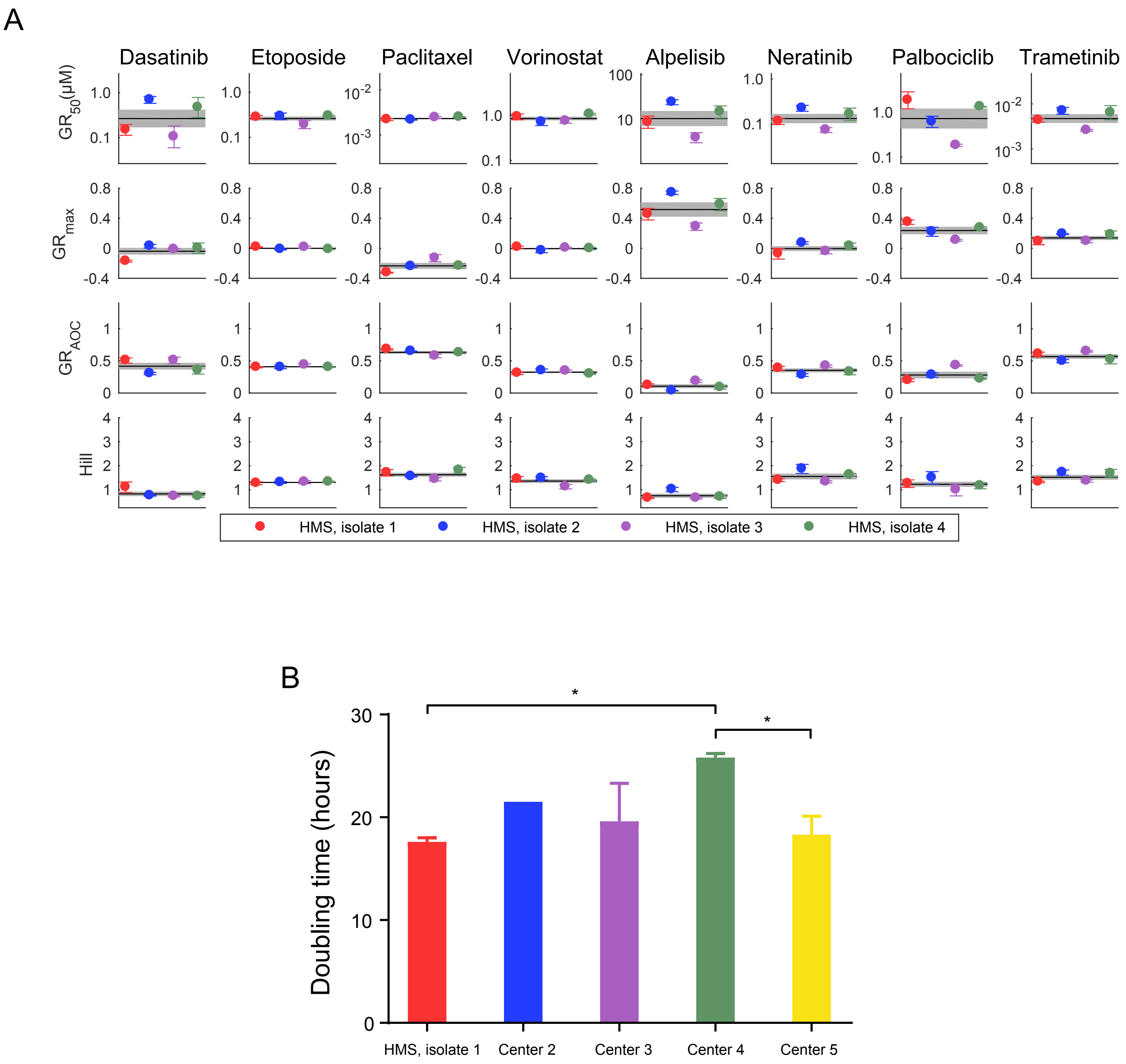

Figure S4, Related to Figure 3

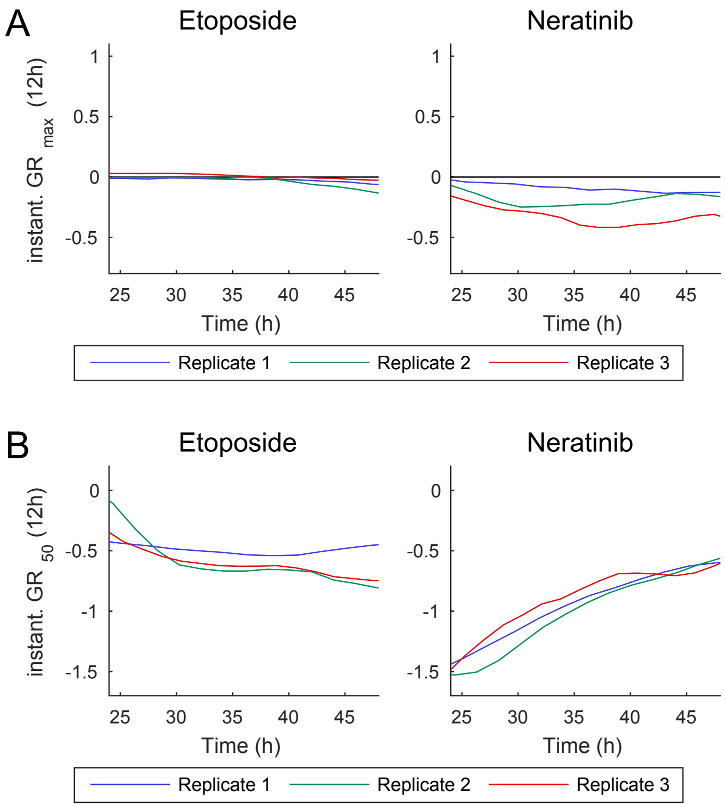

Figure S5, Related to Figure 4

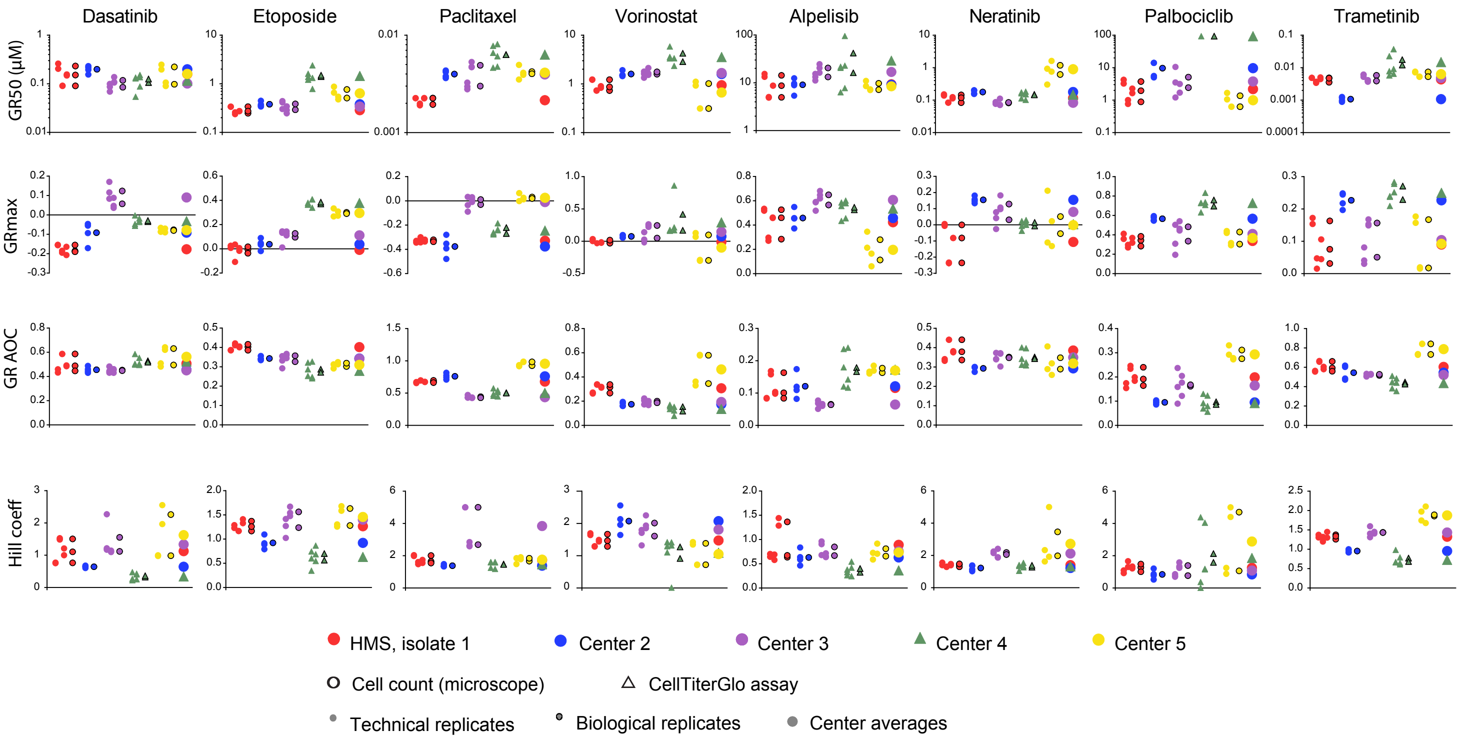

Figure S6, Related to Figure 6

Supplemental Figure Legends

Figure S1, Related to Figure 1: GR dose-response curve and metrics

Schematic of a dose-response curve under the GR model and the source of the derived metrics.

Figure S2, Related to Figure 2: Assay stability over time

GR metrics of MCF 10A cells treated with each drug showing the mean and standard deviation of biological triplicates collected by an experienced research scientist (Scientist A, circles), by the same scientist two years later (squares), by a new technician two years later (Scientist B, triangles), and biological duplicates collected as part of a separate LINCS project in Center One (http://lincs.hms.harvard.edu/db/datasets/20344/) (Scientist C).

Figure S3, Related to Figure 3: GR metrics describing the initial experiments to assess sensitivity of MCF 10A cells to eight drugs measured at three Centers. Center Three and Center Four represent the final results provided by each Center and are the same data presented in Figure 6A. Preliminary 1 represents initial experiments run by Center Three, which showed poor agreement with data from the other two Centers (see table for pipeline details). The disparate results reflect in part differences in readout (CTG vs. Imaging) for Etoposide, Vorinostat, Alpelisib, and Palbociclib. Preliminary 2 represents a coordinated effort by Centers Three and Four: daughter drug plates used by Center Three in Preliminary 1 experiments were shipped to Center Four for cell culture. For many drugs, there is good agreement between Preliminary 1 and Preliminary 2, indicating consistency between cell culturing. The consistent discrepancy in responses to Trametinib between Preliminary 1-Preliminary 2 studies and Center Three-Center Four studies indicate errors in construction of the drug dilution series.

Figure S4, Related to Figure 3: GR metrics across MCF 10A isolates

(A) GR metrics describing the sensitivity of four different MCF 10A cell isolates to eight drugs measured independently at the HMS LINCS center. The black line shows the mean sensitivity measured across all isolates, and the gray box shows the standard error of the mean. (B) The doubling time of MCF10A cells at each LINCS Center. The error bars represent the standard deviation of the mean, the * indicates $P<0.05$ by one way ANOVA with Tukey's multiple comparison tests.

Figure S5, Related to Figure 4: Instantaneous GR metrics

Instantaneous GR_{max} (A) and GR_{50} (B) values in MCF 10A cells treated with Etoposide (left) and Neratinib (right) over the course of 24 hrs for three biological repeats.

Figure S6, Related to Figure 6: Technical and biological variability in GR metrics across Centers

GR metrics for all technical and biological replicates of MCF 10A cells treated with eight drugs by five LINCS Centers (circles represent data from image-based assays and triangles from CellTiter-Glo® assays).

Supplemental Tables

Table S1, Related to STAR Methods: STR profiling results for all MCF 10A isolates used.

Sample Name	Comment	Marker	Allele 1	Allele 2	Peak Height Allele 1	Peak Height Allele 2
NTC	no template control	TH01	0	0		
NTC	no template control	D21S11	0	0		
NTC	no template control	D5S818	0	0		
NTC	no template control	D13S317	0	0		
NTC	no template control	D7S820	0	0		
NTC	no template control	D16S539	0	0		
NTC	no template control	CSF1PO	0	0		
NTC	no template control	Amelogenin				
NTC	no template control	vWA	0	0		
NTC	no template control	TPOX	0	0		
2800M_pos_ctrl	positive control	TH01	6	9.3	2105	2151
2800M_pos_ctrl	positive control	D21S11	29	31.2	1716	1751
2800M_pos_ctrl	positive control	D5S818	12		6411	
2800M_pos_ctrl	positive control	D13S317	9	11	1919	1757
2800M_pos_ctrl	positive control	D7S820	8	11	2650	2704
2800M_pos_ctrl	positive control	D16S539	9	13	2911	2766
2800M_pos_ctrl	positive control	CSF1PO	12		6539	
2800M_pos_ctrl	positive control	Amelogenin	X	Y	1692	1659
2800M_pos_ctrl	positive control	vWA	16	19	724	696
2800M_pos_ctrl	positive control	TPOX	11		2550	
MCF10A-HMS_1	replicate 1	TH01	8	9.3	309	238
MCF10A-HMS_1	replicate 1	D21S11	28	30	295	259
MCF10A-HMS_1	replicate 1	D5S818	10	13	529	1018
MCF10A-HMS_1	replicate 1	D13S317	8	9	280	349
MCF10A-HMS_1	replicate 1	D7S820	10	11	609	525
MCF10A-HMS_1	replicate 1	D16S539	11	12	358	379
MCF10A-HMS_1	replicate 1	CSF1PO	10	12	496	945
MCF10A-HMS_1	replicate 1	Amelogenin	X		488	
MCF10A-HMS_1	replicate 1	vWA	15	17	132	118
MCF10A-HMS_1	replicate 1	TPOX	9	11	191	155
MCF10A-HMS_2	replicate 2	TH01	8	9.3	294	317
MCF10A-HMS_2	replicate 2	D21S11	28	30	327	298
MCF10A-HMS_2	replicate 2	D5S818	10	13	550	1046
MCF10A-HMS_2	replicate 2	D13S317	8	9	347	314
MCF10A-HMS_2	replicate 2	D7S820	10	11	665	429
MCF10A-HMS_2	replicate 2	D16S539	11	12	532	560
MCF10A-HMS_2	replicate 2	CSF1PO	10	12	568	1096
MCF10A-HMS_2	replicate 2	Amelogenin	X		489	
MCF10A-HMS_2	replicate 2	vWA	15	17	173	112
MCF10A-HMS_2	replicate 2	TPOX	9	11	152	192
MCF10A-H2B-mCherry_1	replicate 1	TH01	8	9.3	288	229
MCF10A-H2B-mCherry_1	replicate 1	D21S11	28	30	272	276
MCF10A-H2B-mCherry_1	replicate 1	D5S818	10	13	363	904
MCF10A-H2B-mCherry_1	replicate 1	D13S317	8	9	205	273

Sample Name	Comment	Marker	Allele 1	Allele 2	Peak Height Allele 1	Peak Height Allele 2
MCF10A-H2B-mCherry_1	replicate 1	D7S820	10	11	341	420
MCF10A-H2B-mCherry_1	replicate 1	D16S539	11	12	400	371
MCF10A-H2B-mCherry_1	replicate 1	CSF1PO	10	12	520	807
MCF10A-H2B-mCherry_1	replicate 1	Amelogenin	X		353	
MCF10A-H2B-mCherry_1	replicate 1	vWA	15	17	104	97
MCF10A-H2B-mCherry_1	replicate 1	TPOX	9	11	140	122
MCF10A-H2B-mCherry_2	replicate 2	TH01	8	9.3	178	183
MCF10A-H2B-mCherry_2	replicate 2	D21S11	28	30	200	197
MCF10A-H2B-mCherry_2	replicate 2	D5S818	10	13	256	495
MCF10A-H2B-mCherry_2	replicate 2	D13S317	8	9	158	227
MCF10A-H2B-mCherry_2	replicate 2	D7S820	10	11	380	374
MCF10A-H2B-mCherry_2	replicate 2	D16S539	11	12	262	265
MCF10A-H2B-mCherry_2	replicate 2	CSF1PO	10	12	283	664
MCF10A-H2B-mCherry_2	replicate 2	Amelogenin	X		230	
MCF10A-H2B-mCherry_2	replicate 2	vWA	15	17	79	76
MCF10A-H2B-mCherry_2	replicate 2	TPOX	9	11	125	94
MCF10A-OHSU_1	replicate 1	TH01	8	9.3	195	236
MCF10A-OHSU_1	replicate 1	D21S11	28	30	236	217
MCF10A-OHSU_1	replicate 1	D5S818	10	13	362	766
MCF10A-OHSU_1	replicate 1	D13S317	8	9	220	257
MCF10A-OHSU_1	replicate 1	D7S820	10	11	430	433
MCF10A-OHSU_1	replicate 1	D16S539	11	12	399	262
MCF10A-OHSU_1	replicate 1	CSF1PO	10	12	428	840
MCF10A-OHSU_1	replicate 1	Amelogenin	X		397	
MCF10A-OHSU_1	replicate 1	vWA	15	17	88	111
MCF10A-OHSU_1	replicate 1	TPOX	9	11	130	125
MCF10A-OHSU_2	replicate 2	TH01	8	9.3	267	261
MCF10A-OHSU_2	replicate 2	D21S11	28	30	291	313
MCF10A-OHSU_2	replicate 2	D5S818	10	13	500	1000
MCF10A-OHSU_2	replicate 2	D13S317	8	9	262	328
MCF10A-OHSU_2	replicate 2	D7S820	10	11	622	407
MCF10A-OHSU_2	replicate 2	D16S539	11	12	396	368
MCF10A-OHSU_2	replicate 2	CSF1PO	10	12	431	789
MCF10A-OHSU_2	replicate 2	Amelogenin	X		485	
MCF10A-OHSU_2	replicate 2	vWA	15	17	157	150
MCF10A-OHSU_2	replicate 2	TPOX	9	11	231	201
MCF10A-GM_1	replicate 1	TH01	8	9.3	256	196
MCF10A-GM_1	replicate 1	D21S11	28	30	297	252
MCF10A-GM_1	replicate 1	D5S818	10	13	628	1394
MCF10A-GM_1	replicate 1	D13S317	8	9	431	366
MCF10A-GM_1	replicate 1	D7S820	10	11	679	463
MCF10A-GM_1	replicate 1	D16S539	11	12	519	497
MCF10A-GM_1	replicate 1	CSF1PO	10	12	654	1266
MCF10A-GM_1	replicate 1	Amelogenin	X		729	
MCF10A-GM_1	replicate 1	vWA	15	17	149	159
MCF10A-GM_1	replicate 1	TPOX	9	11	174	177

Sample Name	Comment	Marker	Allele 1	Allele 2	Peak Height Allele 1	Peak Height Allele 2
MCF10A-GM_2	replicate 2	TH01	8	9.3	452	389
MCF10A-GM_2	replicate 2	D21S11	28	30	505	576
MCF10A-GM_2	replicate 2	D5S818	10	13	943	1395
MCF10A-GM_2	replicate 2	D13S317	8	9	489	461
MCF10A-GM_2	replicate 2	D7S820	10	11	890	755
MCF10A-GM_2	replicate 2	D16S539	11	12	713	591
MCF10A-GM_2	replicate 2	CSF1PO	10	12	676	1415
MCF10A-GM_2	replicate 2	Amelogenin	X		893	
MCF10A-GM_2	replicate 2	vWA	15	17	268	210
MCF10A-GM_2	replicate 2	TPOX	9	11	240	278
